# Biomarkers of inflammation and breast cancer risk: a case-control study nested in the EPIC-Varese cohort

**DOI:** 10.1038/s41598-017-12703-x

**Published:** 2017-10-05

**Authors:** Claudia Agnoli, Sara Grioni, Valeria Pala, Alessandra Allione, Giuseppe Matullo, Cornelia Di Gaetano, Giovanna Tagliabue, Sabina Sieri, Vittorio Krogh

**Affiliations:** 1 Epidemiology and Prevention Unit, Fondazione IRCCS Istituto Nazionale dei Tumori, Milan, Italy; 2 Medical Sciences Department, University of Torino, Torino, Italy; 3 Italian Institute for Genomic Medicine (IIGM), Torino, Italy; 4 Lombardy Cancer Registry Unit, Fondazione IRCCS Istituto Nazionale dei Tumori, Milan, Italy

## Abstract

Breast cancer (BC) is the leading cause of cancer death in women. Adipokines, and other inflammation molecules linked to adiposity, are suspected to be involved in breast carcinogenesis, however prospective findings are inconclusive. In a prospective nested case-control study within the EPIC-Varese cohort, we used conditional logistic regression to estimate rate ratios (RRs) for BC, with 95% confidence intervals (CI), in relation to plasma levels of C-reactive protein (CRP), tumor necrosis factor-alpha (TNF-α), interleukin-6, leptin, and adiponectin, controlling for BC risk factors. After a median 14.9 years, 351 BC cases were identified and matched to 351 controls. No marker was significantly associated with BC risk overall. Significant interactions between menopausal status and CRP, leptin, and adiponectin were found. Among postmenopausal women, high CRP was significantly associated with increased BC risk, and high adiponectin with significantly reduced risk. Among premenopausal women, high TNF-α was associated with significantly increased risk, and high leptin with reduced risk; interleukin-6 was associated with increased risk only in a continuous model. These findings constitute further evidence that inflammation plays a role in breast cancer. Interventions to lower CRP, TNF-α, and interleukin-6 and increase adiponectin levels may contribute to preventing BC.

## Introduction

Breast cancer is the commonest cancer and leading cause of cancer death in women worldwide, with an estimated 1.7 million cases and over 520,000 deaths in 2012, accounting for 25% of all female cancers and 15% of all female cancer deaths^1^.

As long ago as 1863, Rudolf Virchow proposed that cancers originate at sites of chronic inflammation^2^. It is now clear that chronic inflammation is associated with several human cancers and that pro-inflammatory cytokines and other immunomodulatory molecules can be produced by cells in cancerous tissue to favor tumor growth, infiltration and metastasis^3^. C-reactive protein (CRP), an acute-phase protein of hepatic origin that is a sensitive yet nonspecific marker of the inflammatory response, has been associated with breast cancer risk in some^4–7^ but not all studies^6^. Several adipokines (immunomodulatory proteins produced by adipose and other tissues) are also suspected to play a role in breast carcinogenesis^8^. In particular, altered levels of tumor necrosis factor-alpha (TNF-α), interleukin-6 (IL-6), adiponectin, and leptin have important roles in promoting inflammation in obesity and chronic inflammatory diseases^9^, and may play a role in carcinogenesis^8,10^. However prospective studies on markers of inflammation and breast cancer risk have produced conflicting results^11–13^.

We carried out a case-control study to prospectively assess whether pre-diagnostic levels of CRP, TNF-α, IL-6, leptin, and adiponectin in plasma are associated with risk of developing breast cancer.

## Results

Baseline characteristics of cases and controls, by tertiles of plasma level of inflammatory biomarkers, are shown in Table 1. Women in the highest tertiles of all biomarkers tended to be older and have higher BMI (lower BMI for high adiponectin). Alcohol intake decreased with increasing adiponectin levels. Women in the highest tertiles were also less educated (not for increasing levels of adiponectin), less likely to be smokers (not for increasing levels of IL-6) or sometime oral contraceptive users, and more likely to be postmenopausal. Age at menarche was higher for increasing levels of CRP and IL-6. Lastly, while women with highest CRP levels were less likely to be nulliparous, the opposite was the case for women with highest adiponectin levels.

**Table 1:** Baseline characteristics of study participants by tertiles of plasma biomarkers.

	C-reactive protein			TNF-α			Interleukin-6			Leptin			Adiponectin		
	I (n = 237)	II (n = 227)	III (n = 238)	I (n = 236)	II (n = 204)	III (n = 262)	I (n = 227)	II (n = 232)	III (n = 243)	I (n = 243)	II (n = 229)	III (n = 230)	I (n = 242)	II (n = 242)	III (n = 218)
	Mean (SD)														
Age, years	50.0 (8.5)	52.9 (8.3)	53.3 (8.0)	49.8 (9.0)	53.0 (8.3)	53.2 (7.4)	47.3 (7.1)	53.5 (8.5)	55.0 (7.4)	50.3 (8.2)	52.3 (8.4)	53.5 (8.2)	50.7 (7.8)	52.2 (8.1)	53.2 (9.1)
Body mass index, kg/m^2^	23.9 (4.2)	25.8 (3.6)	28.1 (5.3)	25.4 (4.6)	26.3 (4.2)	26.1 (5.2)	24.3 (3.5)	26.2 (4.9)	27.2 (5.2)	22.7 (2.5)	25.9 (4.3)	29.4 (4.5)	26.9 (5.0)	25.7 (5.1)	25.1 (3.7)
Alcohol consumption, g/d	8.5 (11.3)	9.2 (12.3)	6.8 (10.9	7.7 (11.1)	7.0 (10.5)	9.4 (12.5)	7.5 (10.9)	8.5 (11.6)	8.5 (12.0)	9.4 (12.3)	7.5 (10.8)	7.5 (11.3)	8.7 (12.6)	8.8 (11.4)	6.8 (10.2)
Plasma C-reactive protein, ng/ml	1150 (566)	3497 (998)	16601 (21101)	6637 (11530	6467 (12811)	8138 (16801)	4100 (6354)	6925 (14580)	10207 (17724)	3564 (4829)	6937 (14593)	11143 (18414)	8217 (16693)	6588 (10909)	6582 (13977)
Plasma TNF-α, pg/ml	2.3 (8.0)	1.9 (3.7)	1.9 (7.2)	0.2 (0.2)	1.1 (0.3)	4.4 (10.4)	0.6 (0.7)	1.4 (1.0)	4.1 (10.9	1.9 (3.5)	2.1 (7.5)	2.2 (8.0)	2.1 (5.9)	2.4 (8.0)	1.7 (5.6)
Plasma interleukin-6, pg/ml	2.6 (3.6)	3.3 (4.9)	4.1 (5.8)	1.7 (2.3)	2.8 (1.7)	5.2 (7.1)	0.8 (0.5)	2.5 (0.5)	6.5 (7.2)	2.8 (4.6)	3.4 (6.0)	3.8 (4.1)	3.5 (3.4)	3.7 (7.1)	2.7 (2.9)
Plasma leptin, pg/ml	5189 (3711)	7584 (4192)	9868 (5406)	7718 (5211	7764 (4632)	7232 (4783)	6373 (4242)	7661 (4542)	8926 (5425)	2911 (1056)	6619 (1225)	13378 (3557)	8634 (5241)	7218 (5094)	6715 (3965)
	N (%)														
**Age at menarche**: < 15 years	216 (91%)	202 (89%)	206 (87%)	205 (87%)	185 (91%)	234 (89%)	210 (93%)	204 (88%)	210 (86%)	216 (89%)	201 (88%)	207 (90%)	220 (92%)	208 (86%)	194 (89%)
≥15 years	21 (9%)	25 (11%)	32 (13%)	31 (13%)	19 (9%)	28 (11%)	17 (7%)	28 (12%)	33 (14%)	27 (11%)	28 (12%)	23 (10%)	20 (8%)	34 (14%)	24 (11%)
**Menopausal status**: postmenopausal	85 (36%)	121 (53%)	128 (54%)	84 (36%)	99 (49%)	151 (58%)	51 (22%)	125 (54%)	158 (65%)	95 (39%)	118 (52%)	121 (53%)	98 (40%)	117 (48%)	119 (55%)
premenopausal	148 (62%)	105 (46%)	107 (45%)	146 (62%)	103 (50%)	111 (42%)	170 (75%)	105 (45%)	85 (35%)	146 (60%)	108 (47%)	106 (46%)	143 (59%)	123 (51%)	94 (43%)
perimenopausal	4 (2%)	1 (1%)	3 (1%)	6 (2%)	2 (1%)	0 (0%)	6 (3%)	2 (1%)	0 (0%)	2 (1%)	3 (2%)	3 (1%)	1 (1%)	2 (1%)	5 (3%)
**Parity**: nulliparous	38 (16%)	22 (10%)	15 (6%)	25 (10%)	25 (12%)	25 (10%)	31 (14%)	21 (9%)	23 (10%)	24 (10%)	26 (11%)	25 (11%)	20 (8%)	22 (9%)	33 (15%)
1–2 children	161 (68%)	159 (70%)	162 (68%)	169 (72%)	132 (65%)	181 (69%)	167 (73%)	161 (69%)	154 (63%)	178 (73%)	160 (70%)	144 (63%)	167 (69%)	168 (69%)	147 (67%)
>2 children	38 (16%)	46 (20%)	61 (26%)	42 (18%)	47 (23%)	56 (21%)	29 (13%)	50 (22%)	66 (27%)	41 (17%)	43 (19%)	61 (26%)	55 (23%)	52 (22%)	38 (18%)
**Oral contraceptive use**: never	134 (57%)	141 (62%)	150 (63%)	126 (53%)	124 (61%)	175 (67%)	105 (46%)	148 (64%)	172 (71%)	138 (57%)	131 (57%)	156 (68%)	140 (58%)	145 (60%)	140 (64%)
ever	103 (43%)	86 (38%)	88 (37%)	110 (47%)	80 (39%)	87 (33%)	122 (54%)	84 (36%)	71 (29%)	105 (43%)	98 (43%)	74 (32%)	102 (42%)	97 (40%)	78 (36%)
**Education**: ≤8 years	73 (31%)	93 (41%)	110 (46%)	65 (28%)	95 (47%)	116 (44%)	54 (24%)	100 (43%)	122 (50%)	81 (33%)	83 (36%)	112 (49%)	100 (41%)	96 (40%)	80 (37%)
>8 years	164 (69%)	134 (59%)	128 (54%)	171 (72%)	109 (53%)	146 (56%)	173 (76%)	132 (57%)	121 (50%)	162 (67%)	146 (64%)	118 (51%)	142 (59%)	146 (60%)	138 (63%)
**Smoking status**: current smoker	52 (22%)	29 (13%)	38 (16%)	51 (22%)	22 (11%)	46 (17%)	40 (18%)	33 (14%)	46 (19%)	61 (25%)	31 (14%)	27 (12%)	44 (18%)	41 (17%)	34 (16%)
ex-smoker	44 (19%)	34 (15%)	33 (14%)	43 (18%)	27 (13%)	41 (16%)	49 (21%)	32 (14%)	30 (12%)	31 (13%)	48 (20%)	32 (14%)	36 (15%)	39 (16%)	36 (16%)
never smoker	141 (59%)	164 (72%)	167 (70%)	142 (60%)	155 (76%)	175 (67%)	138 (61%)	167 (72%)	167 (69%)	151 (62%)	150 (66%)	171 (74%)	162 (67%)	162 (67%)	148 (68%)

Table 2 shows RRs of developing breast cancer by tertiles of plasma markers. None of the markers was significantly associated with risk. Table 3 shows results for postmenopausal and premenopausal women separately. Significant interactions between menopausal status and plasma levels were found for CRP (tertiles model), leptin (tertiles and continuous models), and adiponectin (tertiles model).

**Table 2:** Rate ratios (RR) for developing breast cancer in relation to tertiles of plasma levels of inflammation biomarkers.

	Tertile I	Tertile II	Tertile III	P trend	Continuous (for 1 SD* increase)
**C-reactive protein**					
Range, ng/ml	0.005–2045.97	2047.44–5462.91	5469.28–164231.26		
Cases/Controls	120/117	110/117	121/117		
RR^1^ (95% CI**)	1	0.89 (0.62–1.289	1.06 (0.70–1.59)	0.843	0.91 (0.76–1.09)
RR^2^ (95% CI)	1	0.95 (0.65–1.38)	1.15 (0.75–1.76)	0.553	0.91 (0.76–1.08)
**TNF-α**					
Range, pg/ml	0.009–0.6	0.605–1.63	1.64–110.51		
Cases/Controls	117/119	89/115	145/117		
RR^1^ (95% CI)	1	0.83 (0.50–1.38)	1.36 (0.81–2.30)	0.093	1.25 (0.97–1.61)
RR^2^ (95% CI)	1	0.84 (0.50–1.43)	1.36 (0.79–2.34)	0.105	1.27 (0.97–1.65)
**Interleukin-6**					
Range, pg/ml	0.06–1.64	1.65–3.39	3.41–81.97		
Cases/Controls	110/117	115/117	126/117		
RR^1^ (95% CI)	1	1.15 (0.71–1.84)	1.40 (0.80–2.45)	0.224	1.15 (0.95–1.39)
RR^2^ (95% CI)	1	1.22 (0.75–1.98)	1.58 (0.89–2.82)	0.114	1.17 (0.96–1.43)
**Leptin**					
Range, pg/ml	222.82–4662.34	4672.62–8946.89	8949.08–26775.92		
Cases/Controls	126/117	112/117	113/117		
RR^1^ (95% CI)	1	0.81 (0.54–1.21)	0.83 (0.52–1.34)	0.440	0.96 (0.77–1.19)
RR^2^ (95% CI)	1	0.78 (0.51–1.19)	0.83 (0.51–1.37)	0.486	0.98 (0.78–1.22)
**Adiponectin**					
Range, pg/ml	976575.38–6567600	6590400–10776000	10778000–60520000		
Cases/Controls	125/117	125/117	101/117		
RR^1^ (95% CI)	1	0.98 (0.67–1.43)	0.72 (0.48–1.09)	0.126	0.88 (0.74–1.04)
RR^2^ (95% CI)	1	1.05 (0.71–1.55)	0.73 (0.48–1.11)	0.155	0.89 (0.74–1.05)

*Standard deviation; **Confidence interval

^1^Adjusted for age and BMI.

^2^Further adjusted for family history of breast cancer, age at menarche, parity, oral contraceptive use, smoking status, education, and alcohol consumption.

**Table 3:** Rate ratios (RR) for developing breast cancer in relation to tertiles of plasma levels of inflammation biomarkers by menopausal status.

	Tertile I	Tertile II	Tertile III	P trend	Continuous (for 1 SD* increase)
**Postmenopausal women**					
**C-reactive protein**					
Range, ng/ml	0.005–2045.97	2047.44–5462.91	5469.28–164231.26		
Cases/Controls	36/49	62/59	69/59		
RR^1^ (95% CI**)	1	1.45 (0.80–2.64)	1.77 (0.93–3.35)	0.087	0.77 (0.51–1.15)
RR^2^ (95% CI)	1	1.70 (0.89–3.23)	**2.42 (1.17**–**5.00)**	**0.018**	0.76 (0.52–1.12)
**TNF-α**					
Range, pg/ml	0.009–0.6	0.605–1.63	1.64–110.51		
Cases/Controls	42/42	48/51	77/74		
RR^1^ (95% CI)	1	0.89 (0.43–1.84)	0.92 (0.44–1.92)	0.869	1.13 (0.90–1.41)
RR^2^ (95% CI)	1	0.85 (0.40–1.81)	0.86 (0.39–1.89)	0.769	1.12 (0.89–1.41)
**Interleukin-6**					
Range, pg/ml	0.06–1.64	1.65–3.39	3.41–81.97		
Cases/Controls	25/26	62/63	80/78		
RR^1^ (95% CI)	1	1.02 (0.45–2.29)	1.19 (0.49–2.88)	0.611	1.07 (0.89–1.28)
RR^2^ (95% CI)	1	1.24 (0.52–3.00)	1.53 (0.59–3.96)	0.345	1.09 (0.90–1.31)
**Leptin**					
Range, pg/ml	222.82–4662.34	4672.62–8946.89	8949.08–26775.92		
Cases/Controls	38/57	67/51	62/59		
RR^1^ (95% CI)	1	**1.84 (1.01**–**3.37)**	1.89 (0.92–3.85)	0.092	1.33 (0.97–1.84)
RR^2^ (95% CI)	1	1.69 (0.89–3.19)	1.74 (0.83–3.63)	0.164	1.30 (0.94–1.81)
**Adiponectin**					
Range, pg/ml	976575.38–6567600	6590400–10776000	10778000–60520000		
Cases/Controls	59/39	55/62	53/66		
RR^1^ (95% CI)	1	**0.49 (0.26**–**0.91**	**0.39 (0.21**–**0.76)**	**0.006**	0.81 (0.63–1.04)
RR^2^ (95% CI)	1	**0.49 (0.25**–**0.95)**	**0.37 (0.19**–**0.72)**	**0.004**	0.80 (0.62–1.04)
**Premenopausal women**					
**C-reactive protein**					
Range, ng/ml	0.005–2045.97	2047.44–5462.91	5469.28–164231.26		
Cases/Controls	82/66	47/58	51/56		
RR^1^ (95% CI)	1	0.68 (0.42–1.09)	0.72 (0.40–1.29)	0.171	0.94 (0.77–1.15)
RR^2^ (95% CI)	1	0.68 (0.41–1.14)	0.74 (0.40–1.37)	0.253	0.94 (0.76–1.16)
**TNF-α**					
Range, pg/ml	0.009–0.6	0.605–1.63	1.64–110.51		
Cases/Controls	72/74	40/63	68/43		
RR^1^ (95% CI)	1	0.76 (0.36–1.62)	2.07 (0.94–4.57)	**0.016**	1.78 (0.89–3.55)
RR^2^ (95% CI)	1	0.80 (0.36–1.76)	2.15 (0.95–4.86)	**0.017**	1.81 (0.91–3.61)
**Interleukin-6**					
Range, pg/ml	0.06–1.64	1.65–3.39	3.41–81.97		
Cases/Controls	82/88	52/53	46/39		
RR^1^ (95% CI)	1	1.19 (0.65–2.18)	1.65 (0.76–3.59)	0.212	1.49 (0.96–2.31)
RR^2^ (95% CI)	1	1.25 (0.67–2.33)	1.89 (0.83–4.28)	0.136	**1.58 (1.02**–**2.46)**
**Leptin**					
Range, pg/ml	222.82–4662.34	4672.62–8946.89	8949.08–26775.92		
Cases/Controls	87/59	43/65	50/56		
RR^1^ (95% CI)	1	**0.34 (0.18**–**0.64)**	**0.42 (0.21**–**0.84)**	**0.011**	**0.68 (0.47**–**0.94)**
RR^2^ (95% CI)	1	**0.32 (0.16**–**0.64)**	**0.43 (0.20**–**0.89)**	**0.025**	**0.71 (0.50**–**0.99)**
**Adiponectin**					
Range, pg/ml	976575.38–6567600	6590400–10776000	10778000–60520000		
Cases/Controls	65/78	69/54	46/48		
RR^1^ (95% CI)	1	1.56 (0.94–2.61)	1.09 (0.61–1.94)	0.527	1.02 (0.78–1.35)
RR^2^ (95% CI)	1	1.66 (0.97–2.85)	1.11 (0.61–2.03)	0.486	1.05 (0.79–1.40)

*Standard deviation; **Confidence interval.

^1^Adjusted for age and BMI.

^2^Further adjusted for family history of breast cancer, age at menarche, parity, oral contraceptive use, smoking status, education, and alcohol consumption.

Among postmenopausal women, high levels (third tertile) of CRP were associated with significantly increased risk (RR 2.42; 95% CI: 1.17–5.00) compared to the first tertile, fully-adjusted model; while high levels (third tertile) of adiponectin were associated with significantly reduced risk (RR 0.37; 95% CI: 0.19–0.72) compared to the first tertile, fully-adjusted model. None of the other biomarkers was significantly associated with breast cancer risk in postmenopausal women.

Among premenopausal women, high TNF-α was associated with significantly increased breast cancer risk in the tertile model only (P trend = 0.017); and high IL-6 was associated with increased risk in the continuous model only (RR 1.58; 95% CI: 1.02–2.46). By contrast, high plasma leptin was associated with significantly reduced risk, both in the tertile-based model (RR 0.43; 95% CI: 0.20–0.89, third vs. first tertile) and for a 1 standard deviation increase in leptin (RR 0.71; 95% CI: 0.50–0.99). None of the other biomarkers was significantly associated with breast cancer risk premenopausal women.

## Discussion

In this nested case-control study, none of the inflammatory biomarkers analyzed was associated with breast cancer risk in the overall population. However, there were significant interactions between menopausal status and levels of CRP, leptin, and adiponectin. Among postmenopausal women, high CRP was associated with increased breast cancer risk, and high adiponectin was associated with decreased risk. And among premenopausal women, high TNF-α and IL-6 were associated with increased risk, and high leptin was associated with decreased risk.

Our finding of a direct association between plasma CRP and risk of postmenopausal breast cancer is in line with the findings of two recently published meta-analyses^4,14^. The first^4^, which examined 12 prospective studies, found that risk increased significantly by 7% overall and by 6% in postmenopausal women, for each doubling of CRP concentration. The other study^14^ analyzed 15 cohort and case-control studies, and included premenopausal women, finding that risk increased by 16% for each natural log unit increase in CRP; however when postmenopausal and premenopausal women were analyzed separately, the risk increase was significant only in postmenopausal women.

CRP is an established systemic marker of inflammation, being produced by the liver in response to cytokines (including IL-6 and TNF-α) produced by cells in inflamed tissue^15,16^. The lack of association between CRP and breast cancer risk in premenopausal women suggests that inflammation plays little or no role in premenopausal breast cancer.

However this conclusion is opposed by our finding that high TNF-α was associated with increased breast cancer risk in premenopausal women. Our finding, in fact, contrasts with the results of the only four prospective studies we are aware of to have investigated TNF-α and breast cancer risk^12,17,18^: three found no significant association between TNF-α and breast cancer; while a fourth case-control study on postmenopausal women nested in the Malmö Diet and Cancer Cohort^19^ found an association between high TNF-α and reduced breast cancer risk.

TNF-α is a major mediator of inflammation: its induction, for example by tissue damage, induces a cascade of other inflammatory cytokines, chemokines, growth factors and endothelial adhesins which recruit and activate a range of cells at the site of tissue damage to promote healing^20,21^. However, when produced chronically, TNF-α seems to act as a tumor promoter, contributing to the tissue remodeling and stromal development necessary for tumor growth and spread^20,21^. Recent data suggest that TNF-α is involved in carcinogenesis at least in part because it activates nuclear factor kappa-light-chain-enhancer of activated B cells (NF-κB) (reviewed in^22^), which is responsible for inducing the expression of genes associated with cell proliferation, apoptosis, inflammation, metastasis, and angiogenesis^23^. TNF-α can also stimulate the activity of the inducible nitric oxide synthase (iNOS), which is implicated in cellular changes leading to malignancy (transformation of normal cells, growth of transformed cells, angiogenesis and metastasis of malignant cells^24^).

Although our finding that TNF-α is associated with increased breast cancer risk in premenopausal women is not supported by previous studies, the association could be real since TNF-α has been found to stimulate the enzymes of estrogen synthesis^25^. High TNF-α could therefore promote breast cancer by this mechanism, which is likely to be more important in premenopause − before the fall in estrogen synthesis heralded by the menopause.

We found that plasma IL-6 was associated with increased breast cancer risk among premenopausal women, but only in the continuous model. IL-6 has been reported to activate Janus kinase (JAK) and signal transducer and activator of transcription 3 (STAT3) pathways^22^ to promote a cellular microenvironment that may promote cancer growth. However, no association between increased IL-6 levels and breast cancer risk has been reported in previous studies, specifically the case-cohort study nested within the Women’s Health Initiative Observational Study^12^, the British Women’s Heart and Health Study cohort study and Caerphilly Cohort^26^, and the Health Aging and Body Composition prospective cohort study^17^.

The inverse association we found between plasma leptin and breast cancer risk in premenopausal women is supported by the findings of a case-control study by Harris *et al*.^27^ on premenopausal women. By contrast the case-control study of Touvier *et al*.^13^ found no association between leptin and breast cancer risk. Other prospective studies that investigated plasma leptin and breast cancer mainly involved postmenopausal women, and found no association^12,28^ or a direct association^11,29^. Leptin is thought to be involved in promoting breast cancer in obese women by stimulating the conversion of aromatizable androgens (androstenedione and dehydroepiandrosterone) to estradiol. This occurs not only in adipose but also in breast epithelial cells, particularly when levels of circulating estrogens decline, as they do in postmenopausal women^30,31^. This mechanism might explain why high leptin was associated with non-significantly increased breast cancer risk in our postmenopausal women, even after adjusting for BMI. However, in premenopausal women, high leptin may lower breast cancer risk, since leptin is involved in the regulation of ovarian folliculogenesis^32^ and at high levels may reduce follicular estradiol secretion^33^. Very high leptin levels have been reported in women with chronic anovulation^34^, a condition that may be associated with reduced breast cancer risk^35^.

As regards adiponectin, we found decreased breast cancer risk with increasing levels in postmenopausal women, in agreement with the findings of a 2013 meta-analysis that examined 17 observational studies (4 nested case-control studies and 9 case-control studies)^36^, and found no association of adiponectin with breast cancer risk overall, but decreased risk in postmenopausal women. A 2014 meta-analysis^37^ which examined 15 observational studies (6 prospective and 9 case-control studies), found a 5% reduction in overall breast cancer risk for 3 μg/ml increments in adiponectin, but no significant associations in post- or premenopausal women examined separately. A 2015 case-cohort study on postmenopausal women found no association between plasma adiponectin and breast cancer risk^12^. A 2016 meta-analysis of 107 epidemiological studies^38^ found that circulating adiponectin levels were lower in patients with various cancers than controls. However, other studies have found that increased adiponectin levels correlate with cancer progression (reviewed in^39^); and in patients with viral infections or chronic inflammation, increased levels of adiponectin predict cancer development^39^. Obesity is protective against breast cancer in premenopause but increases breast cancer risk in postmenopause. Adiponectin levels are low in obesity, so a presumed cancer-promoting effect of low adiponectin in premenopause may be masked by concomitant and protective obesity. In postmenopause, obesity is not protective so low levels of adiponectin may be “freed” to exert a cancer promoting effect, possibly explaining our finding that high adiponectin was associated with lowered breast cancer risk in postmenopausal women only^40^.

Strengths of our study are its prospective design, relatively large sample size, and availability of detailed information on lifestyle that made it possible to control for confounding effects. A limitation is that we assessed variables at baseline only and do not know to what extent they may have changed subsequently. Limited data indicate that adipokine levels in a single blood sample are useful biomarkers of inflammation in population-based studies^41^.

Another limitation is that the relation between the circulating levels of the biomarkers we examined and their activity in breast or adipose is unknown, and it is possible that plasma levels may be a poor surrogate for local activity. For example leptin and adiponectin seem to function primarily in a paracrine manner, so circulating levels are unlikely to reflect biological activity in the breast^42^. Another possible limitation is that samples were collected, stored at −196 °C, and analyzed up to 20 years later. There may have been differential decay of the analytes over that period^43,44^. However, unless analyte decay varied with initial concentration (which seems unlikely), this will not bias analyte-risk associations.

To conclude, the findings of this case-control study nested in the EPIC-Varese cohort suggest that high levels of CRP and low levels of adiponectin may increase the risk of postmenopausal breast cancer, while high levels of TNF-α and IL-6, and low levels of leptin may increase breast cancer risk among premenopausal women. Further research is required to elucidate the mechanisms by which leptin can influence the etiology of premenopausal breast cancer; interventions to lower CRP and increase adiponectin levels might help reduce the risk of developing postmenopausal breast cancer, while interventions to lower TNF-α and IL-6 levels might help reduce the risk of developing premenopausal breast cancer.

## Methods

### Study population and data collection

The case-control study was nested within the 9378 women, resident in Varese Province, northern Italy, who were recruited in 1992-1997 (age 35–69 years) to the European Prospective Investigation into Cancer and Nutrition (EPIC)-Varese study, and gave blood samples on recruitment.

At baseline, detailed information was collected on reproductive and medical history, physical activity, alcohol consumption, smoking, education, and socioeconomic variables, using a standardized lifestyle questionnaire. Diet over the previous year was investigated using a food frequency questionnaire specifically developed to capture local dietary habits. Weight, height, and blood pressure were measured, and a 30 mL fasting blood sample was collected. The blood samples were divided into 0.5 mL aliquots of plasma, serum, red blood cells, and buffy coat, on the day of collection, and stored in liquid nitrogen at −196 °C^45^.

### Ethics Statement

The study protocol was approved by the ethics committee of the Fondazione IRCCS Istituto Nazionale dei Tumori, Milan, Italy. At baseline, participants signed a written informed consent to use clinical data for research. Consent forms were stored with barcode ID for subject identification. The ethics committee approved this consent procedure. The study protocol and informed consent procedure met the requirements of Italian legislation and the Declaration of Helsinki of 1975, as revised in 2008.

### Breast cancer cases and selection of control women

The women were followed-up to December 31, 2009 (median 14.9 years), through the Varese section of the Lombardy Cancer Registry, characterized by high data completeness and quality. A total of 362 new breast cancers was identified.

For each case, one matched control was chosen, using an incidence density sampling protocol, from appropriate risk sets consisting of cohort members alive and free of cancer at the time of diagnosis of the index case. Matching criteria were age at recruitment (±5 years), date of recruitment (±180 days), menopausal status (postmenopausal, premenopausal, and perimenopausal at baseline), and analysis of inflammatory markers in the same batch.

### Analysis of plasma samples

Plasma samples were analyzed using Luminex multiplex technology, which determines multiple analytes in a single microwell plate, using antibody kits purchased from Bio-Rad (Bio-Plex, TNF-α, IL-6, leptin, and adiponectin) or Merck (CRP)^46^. All the analyses were performed in duplicated and results with a intra-assay %CV > 20% were discarded. The contents of each well were read by Bio-Plex 100 System array reader (Bio-Rad Laboratories, California, USA), which identifies and quantifies each analyte based on bead color and fluorescent signal intensity. Instrument calibration procedure was performed daily by Bio-Plex Calibration Kit (Bio-Rad) for optimal performance and reproducibility of results. The data were processed using Bio-Plex Manager software (version 6.1) using five-parametric curve fitting and converted to pg/ml.

All kits supplied lyophilized standards that were reconstituted and diluted at 7 serial concentrations following manufacturer’s instructions (standard curves). Standards included all recombinant proteins tested and were considered as positive controls for the procedure. Standard diluent buffers alone were used as negative controls.

### Statistical methods

Plasma levels of inflammatory molecules were grouped into tertiles based on the distribution in controls. Baseline characteristics of cases and controls, according to tertiles of plasma inflammatory biomarkers, were summarized as means and standard deviations (continuous variables) or frequencies (categorical variables). Conditional logistic regression models were used to estimate rate ratios (RRs) for breast cancer with 95% confidence intervals (CIs), with lowest tertile as reference. The significance of linear trends was assessed by treating each tertile as a continuous variable in the model and performing the Wald test. RRs were also calculated for 1 standard deviation increments in plasma concentration as a continuous variable. We ran a minimally adjusted model, adjusted for age (continuous) and BMI (<25 kg/m^2^, 25- < 30 kg/m^2^, ≥30 kg/m^2^), and a fully-adjusted model, with the following additional covariates: age at menarche (<15 years, ≥15 years), parity (nulliparous, 1–2 children, >2 children), oral contraceptive use (never, sometime), education (≤8 years, >8 years), smoking status (never, former, current), and alcohol consumption (continuous).

We analyzed all women, and postmenopausal and premenopausal women separately. *P* values for interaction of inflammatory markers with menopausal status were estimated by adding the product of tertile of plasma concentrations and menopausal status to the model and applying the Wald test.

We excluded seven cases and their matched controls because a plasma sample was not available for the case or the control. We excluded four additional cases and controls because confounder variables were missing for the case or control. The analyses were therefore performed on 351 cases and 351 matched controls, total 702 women – 334 postmenopausal, 360 premenopausal, and 8 perimenopausal. All statistical tests were two-sided, differences were considered significant for *P* < 0.05. The analyses were performed with Stata version 14.0 (College Station, TX, USA).

### Data availability

The data that support the findings of this study are held by the corresponding author, however their availability is restricted: for ethical reasons, the Ethical Committee does not allow open/public sharing of data pertaining to individuals. However aggregated data are available to other researchers, upon request.
